# Characteristics and prognostic factors of adult patients with osteosarcoma from the SEER database

**DOI:** 10.1097/MD.0000000000033653

**Published:** 2023-09-15

**Authors:** Guanghua Deng, Pingbo Chen

**Affiliations:** a Ya’an Hospital of Traditional Chinese Medicine, Department of Orthopedics, Ya’an, China; b The Fourth Affiliated Hospital of Xinjiang Medical University, Department of Orthopedics, Urumqi, China.

**Keywords:** adult, nomogram, osteosarcoma, SEER database

## Abstract

Osteosarcoma is the most common bone malignancy. There are many studies on the prognostic factors of children and adolescents, but the characteristics and prognostic factors of adult osteosarcoma are rarely studied. The aim of this study was to construct a nomogram for predicting the prognosis of adult osteosarcoma. Information on all osteosarcoma patients aged ≥ 18 years from 2004 to 2015 was downloaded from the surveillance, epidemiology and end results database. A total of 70% of the patients were included in the training set and 30% of the patients were included in the validation set. Univariate log-rank analysis and multivariate cox regression analysis were used to screen independent risk factors affecting the prognosis of adult osteosarcoma. These risk factors were used to construct a nomogram to predict 3-year and 5-year prognosis in adult osteosarcoma. Multivariate cox regression analysis yielded 6 clinicopathological features (age, primary site, tumor size, grade, American Joint Committee on Cancer stage, and surgery) for the prognosis of adult osteosarcoma patients in the training cohort. A nomogram was constructed based on these predictors to assess the prognosis of adult patients with osteosarcoma. Concordance index, receiver operating characteristic and calibration curves analyses also showed satisfactory performance of the nomogram in predicting prognosis. The constructed nomogram is a helpful tool for exactly predicting the prognosis of adult patients with osteosarcoma, which could enable patients to be more accurately managed in clinical practice.

## 1. Introduction

Osteosarcoma is the most common primary bone malignancy, especially in children and adolescents,^[1–3]^ and there have been many studies of osteosarcoma in children and adolescents.^[4,5]^ However few reports describe the characteristics and prognostic factors of adult patients with osteosarcoma. Duffaud F^[6]^ studied the efficacy and safety of regorafenib in adult patients with metastatic osteosarcoma. Stefano Testa^[7]^ studied prognosis and prognostic factors in adult and pediatric patients with osteosarcoma. Osteosarcoma has different characteristics at different ages due to its different gene expression, treatment response, and heterogeneous histological subtypes.^[8–11]^ Therefore, identifying adult patients with osteosarcoma at high risk of mortality can ensure appropriate treatment and can have a major impact on prognosis. As a statistical prediction model, the nomogram represents a graphical pattern in which variables are given scores so that it is easy to obtain event probabilities for individual patients compared to traditional assessment criteria. In recent years, with the increasing demand for individualized medicine in patients with various malignancies and different types of osteosarcoma, this model has been widely used.^[12–16]^ Due to the different clinical and prognostic characteristics between the different age groups, no studies were available to develop a prognostic nomogram for osteosarcoma in adult patients. Therefore, in this study, we aimed to construct and validate a survival nomogram incorporating available clinical characteristics to improve the prognosis of adult osteosarcoma patients in clinical practice.

## 2. Methods

### 2.1. Study population

The information was downloaded from the surveillance, epidemiology, and end results (SEER) database for all adult patients (age ≥ 18 years) with osteosarcoma from 2004 to 2015 (using the International Histological Classification of Tumor Disorders, Third Edition (IDO-O-3) code 9180 to 9187 and 9192 to 9195 for identification).

### 2.2. Data collection

The following variables were identified from the dataset: age at diagnosis (18–39, 40–59, or ≥ 60 years), sex (male or female), race (white, black, or others), marital status (married or other), primary site (Limb or other), tumor size (<50 mm, 50–99 mm or ≥ 100 mm), tumor number (1 primary only or other), grade (I, II, III or IV), American Joint Committee on Cancer (AJCC) stage (I, II, III or IV), tumor stage (local/regional or distant), primary site surgery (yes or no), radiation therapy (yes or no), chemotherapy (yes or no). We further excluded patients with survival of <1 month and unknown variables such as race, marital status, primary site, grade, AJCC stage, and tumor size. We used the caret package in R software (version 4.2.1) to randomize 983 patients in a 7:3 ratio into a training set (70%) and a validation set (30%).

### 2.3. Establishment of cox regression models

Univariate log-rank analysis and multivariate cox regression analysis were performed using SPSS version 26 (IBM, Armonk, NY) to identify independent prognostic factors associated with prognosis in adult osteosarcoma patients. The nomogram was drawn and Concordance index (C-index), receiver operating characteristic (ROC), and calibration curves were calculated using the rms, foreign and survival packages in R version 4.2.1 (R development core team, Vienna, Austria) to evaluate the predictive performance of the nomogram.

### 2.4. Statistical analysis

Counts and percentages were used to describe categorical measures. Chi-square tests were used to compare categorical measures. *P* < .05, the difference was statistically significant.

## 3. Results

### 3.1. Demographic baseline characteristics

**Figure 1.**
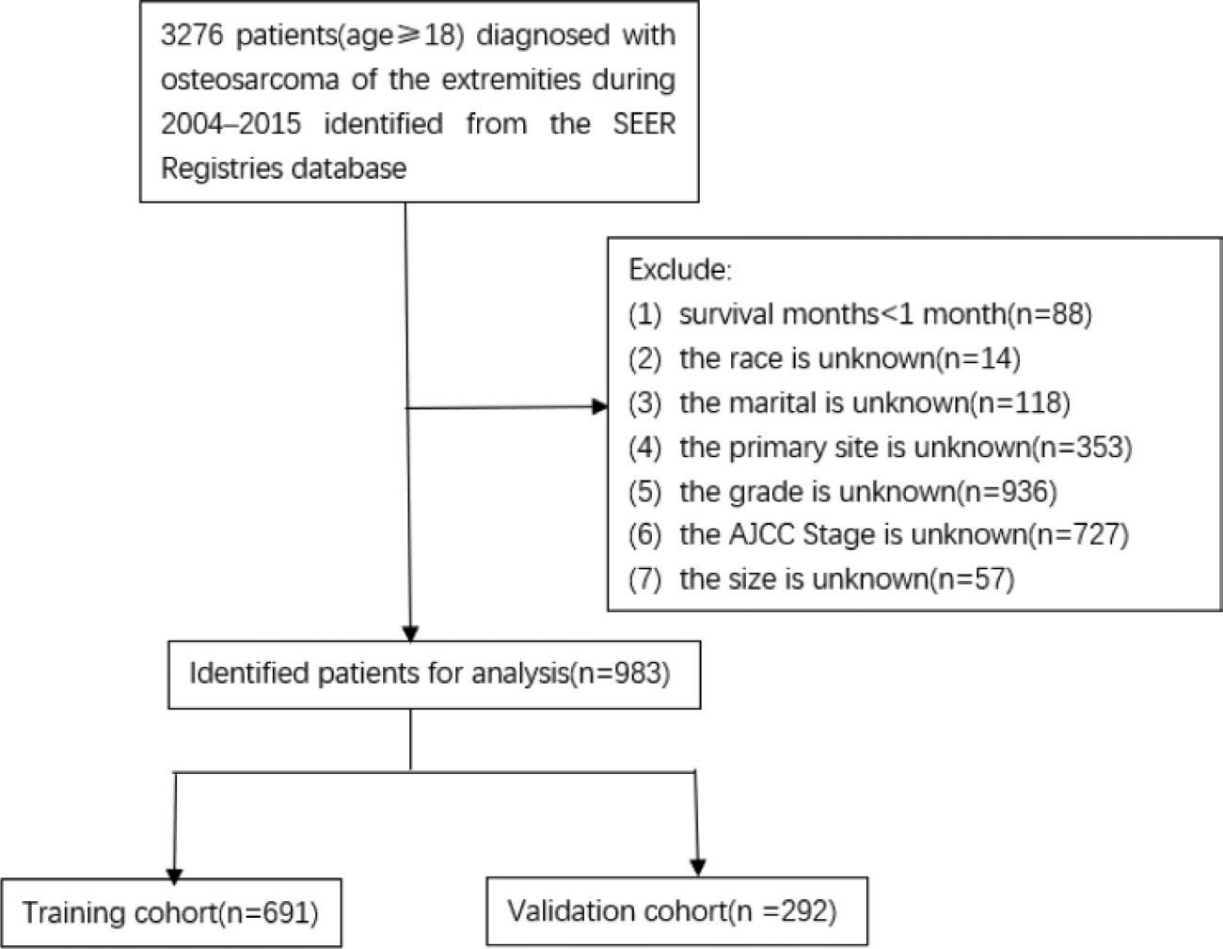
Flowchart of sample selection.

According to the determined inclusion and exclusion criteria, 983 adult patients with osteosarcoma were included from SEER database. We randomly assigned patients to tow sets in R software, 70% of the patients to the training set (n = 691) and 30% to the validation set (n = 292) (Fig. 1). The majority of patients were aged between 18 to 39 (53.3%, 54.1%), White (75.4%, 77.4%), undergone the therapies surgery (87.4%, 90.8%) and Chemotherapy (72.4%, 71.2%), in the training and validation sets, respectively. Table 1 showed the clinicopathologic characteristics of all patients in the training and validation sets.

**Table 1:** Demographics, tumor characteristics, and treatment characteristics of patients with osteosarcoma.

Characteristics	Training set (n = 691)	Test set (n = 292)	*P* value
Age (yr)			.358
18–39	368	158	
40–59	192	70	
≧60	131	64	
Sex			.229
Female	339	131	
Male	352	161	
Race			.780
White	521	226	
Black	98	37	
Other	72	29	
Marital			.720
Married	302	124	
Other	389	168	
Primary site			.873
Limb	458	192	
Other	233	100	
Tumor size (mm)			.697
<50	144	54	
50–99	282	124	
≧100	265	114	
Tumor number			.382
One primary only	561	230	
Other	130	62	
Grade			.813
I	51	23	
II	77	29	
III	206	81	
IV	357	159	
AJCC stage			.770
I	120	48	
II	451	196	
III	13	3	
IV	107	45	
Tumor stage			.244
Location/region	553	243	
Distant	138	49	
Surgery			.135
Yes	604	265	
No	87	27	
Radiation			.761
Yes	92	41	
No	599	251	
Chemotherapy			.719
Yes	500	208	
No	191	84	

AJCC = American Joint Committee on Cancer.

### 3.2. Kaplan–Meier survival analysis and multivariate logistic regression results

**Figure 2.**
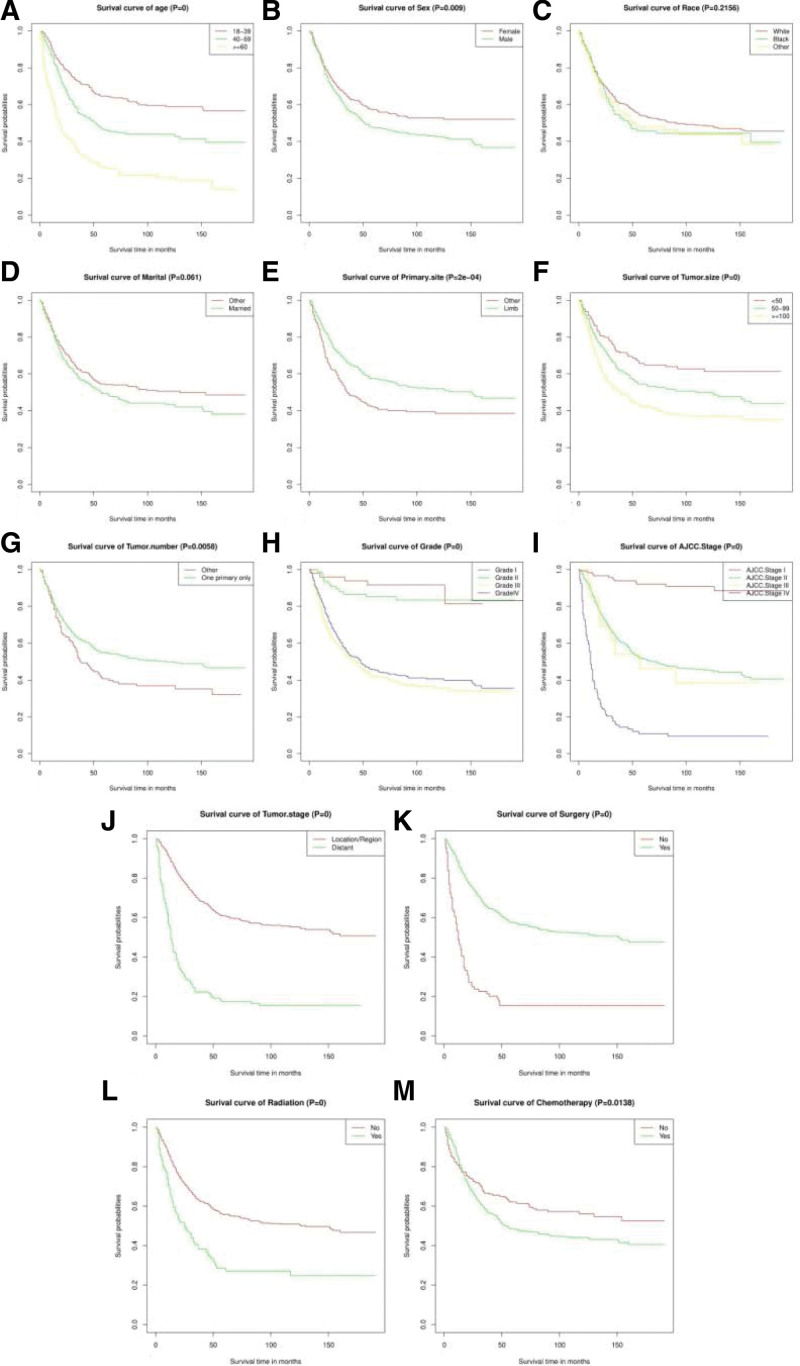
Kaplan–Meier estimated survival in patients with osteosarcoma stratified by age (A), sex (B), race (C), marital (D), primary site (E), tumor size (F), tumor number (G), grade (H), AJCC Stage (I), tumor stage (J), surgery (K), radiation (L), and chemotherapy (M). AJCC = American Joint Committee on Cancer.

According to the Kaplan–Meier survival curves and log-rank tests for categorical variables, race (*P* = .216) and marital (*P* = .061) were not significant factors influencing survival, but others significantly affected patient survival (Fig. 2). In the cox proportional hazards regression model, the hazard ratio (HR) was used for evaluating the relationship between the corresponding variable and patient survival. Age (HR 1.583, 95% CI 1.367–1.832; *P* < .001), primary site (HR 0.682, 95% CI 0.532–0.874; *P* = .003), tumor size (HR 1.315, 95% CI 1.122–1.541; *P* = .001), grade (HR 1.258, 95% CI 1.068–1.482; *P* = .006), AJCC stage (HR 2.129, 95% CI 1.674–2.708; *P* < .001) and surgery (HR 0.529, 95% CI 0.392–0.713; *P* < .001) were significantly related to patient survival (Table 2). But sex (HR 1.143, 95% CI 0.920–1.419; *P* = .227), tumor number (HR 1.021, 95% CI 0.783–1.332; *P* = .877), tumor stage (HR 0.821, 95% CI 0.505–1.334; *P* = .425), radiation (HR 1.119, 95% CI 0.829–1.512; *P* = .462), and chemotherapy (HR 0.863, 95% CI 0.648–1.151; *P* = .317) was not.

**Table 2:** Univariate and multivariate analyses of osteosarcoma.

Risk factors	Univariate analysis	Multivariate analysis		
	*P* value	HR	95% CI	*P* value
Age	<.001	1.583	1.367–1.832	.000
Sex	.009	1.143	0.920–1.419	.227
Race	.216			
Marital	.061			
Primary site	<.001	0.682	0.532–0.874	.003
Tumor size	<.001	1.315	1.122–1.541	.001
Tumor number	.006	1.021	0.783–1.332	.877
Grade	.001	1.258	1.068–1.482	.006
AJCC stage	<.001	2.129	1.674–2.708	.000
Tumor stage	<.001	0.821	0.505–1.334	.425
Surgery	<.001	0.529	0.392–0.713	.000
Radiation	<.001	1.119	0.829–1.512	.462
Chemotherapy	.014	0.863	0.648–1.151	.317

AJCC = American Joint Committee on Cancer, HR = hazard ratio.

### 3.3. Nomogram construction

**Figure 3.**
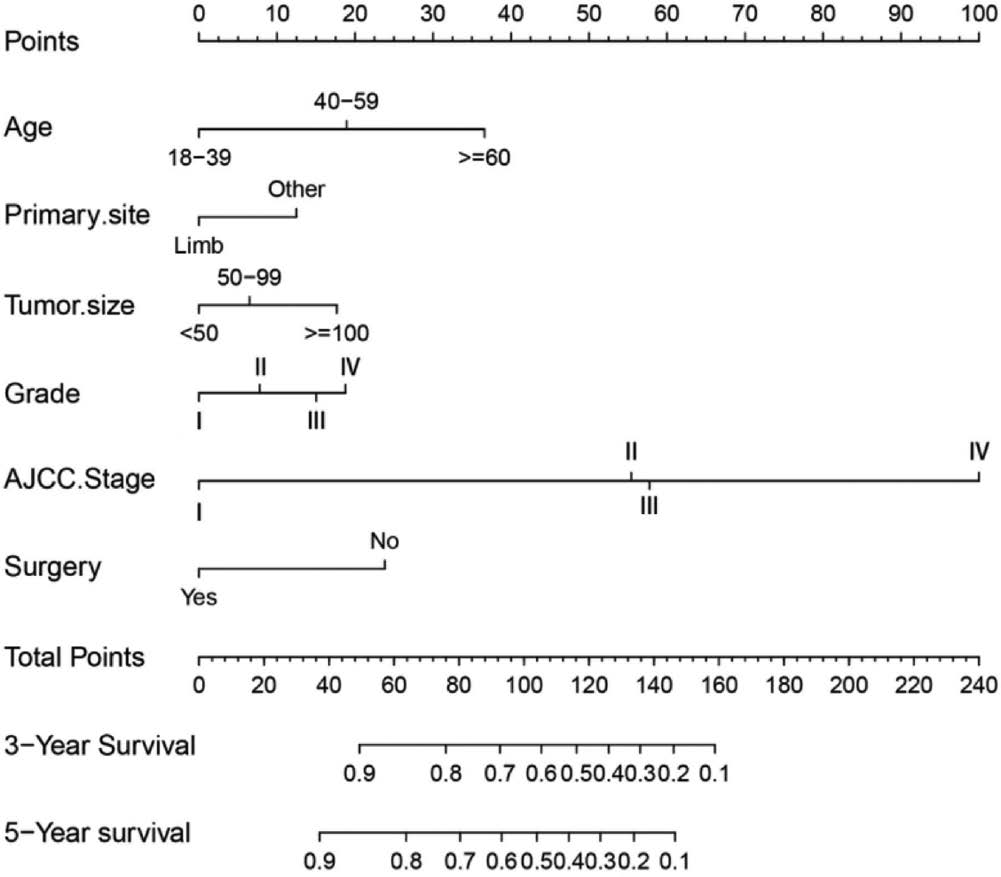
Nomogram to predict 3- and 5-year overall survival for osteosarcoma patients.

The nomogram is a visual regression model. The scoring criteria are set according to the regression coefficients of each influencing factor and the scores of the respective variables are added to obtain the total score of each patient and then calculate the prognosis and survival rate. We included all independent risk factors affecting the prognosis of patients with osteosarcoma, including age, primary site, tumor size, grade, AJCC stage, and surgery, and obtained a rank line that could predict the 3-year and 5-year overall survival (OS) of patients with osteosarcoma picture. The nomogram is mainly composed of variable names and tick marks. The length of the tick marks can reflect the contribution of the influencing factor to the outcome event. The total score can be obtained by adding up the individual scores corresponding to each variable under different values. According to the downward projection of the total score, the survival rate of the corresponding year of the patient can be obtained, as shown in Figure 3.

### 3.4. Validation of the nomogram

**Figure 4.**
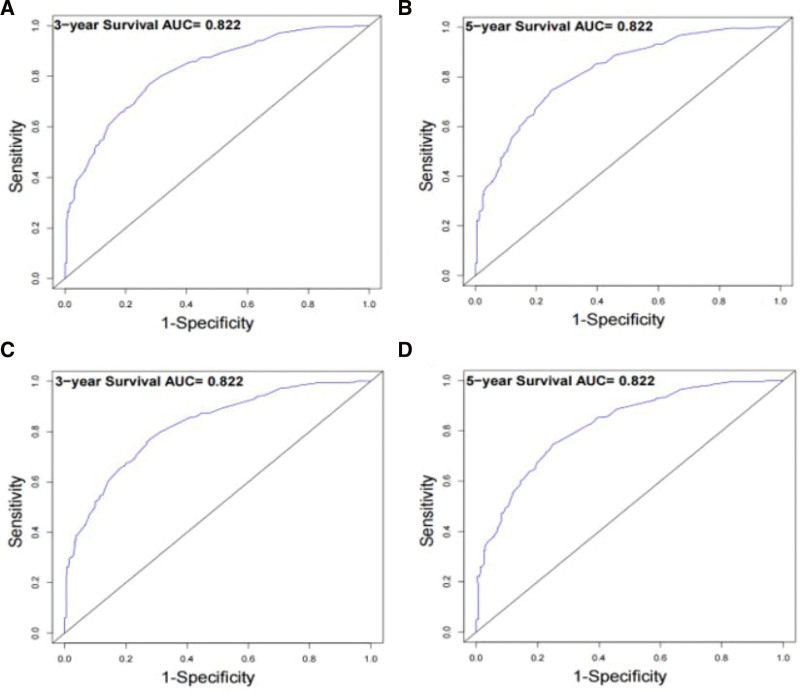
Predictive performance of the survival nomogram reflected by ROC curves. ROC curves for the 3-year and 5-year osteosarcoma in patients in the training cohort (A–B) and in the SEER validation cohort (C–D). ROC = receiver operating characteristic, SEER = surveillance, epidemiology, and end results.

**Figure 5.**
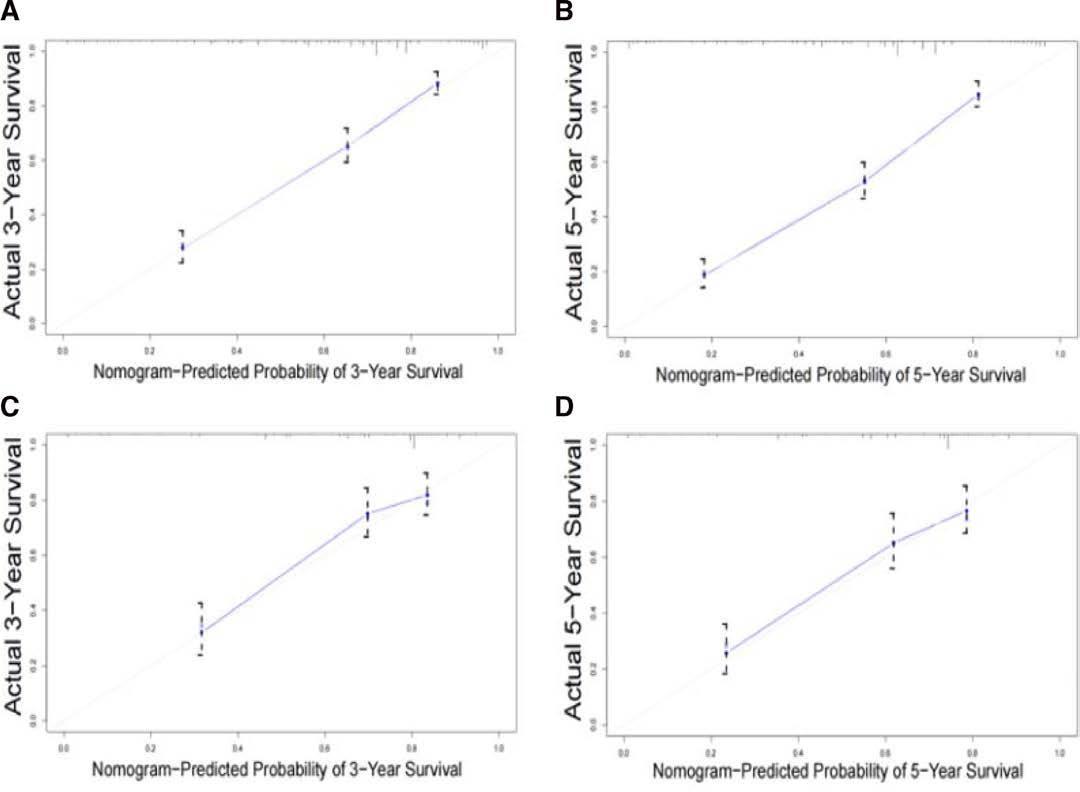
The calibration curves for predicting osteosarcoma in the training and validation sets. Calibration plots of 3-year and 5-year osteosarcoma in the training cohort (A–B) and in the SEER validation cohort (C–D). SEER = surveillance, epidemiology, and end results.

The C-index of the training set is 0.774, and the C-index of the validation set is 0.751, indicating that the model has good accuracy. The areas under the 3-year and 5-year ROC curves of the training set were 0.822 and 0.822, respectively. The areas under the 3-year and 5-year ROC curves of the validation set were 0.822 and 0.822, respectively, as shown in Figure 4. The verification of the calibration curve shows that the model predicts the 3-year and 5-year OS and the actual OS has a good consistency, as shown in Figure 5. Therefore, the established nomogram model is verified by the C-index, ROC curve, and calibration curve to have a good prediction effect.

## 4. Discussion

Prognostic evaluation is of great value for the treatment, monitoring and follow-up of patients with various tumors. As an intuitive and rational prognostic tool, the clinical prediction model has been paid attention to in the prevention and treatment of bone tumors.^[17–20]^

For patients with osteosarcoma, several predictive models have been constructed in previous studies;^[11,13,14]^ however, less attention has been paid to adult osteosarcoma. A study by Wenhao Chen^[21]^concluded that age, tumor site, historical grade, surgery, AJCC T/N, and M were independent prognostic factors for survival in patients with osteosarcoma. However, based on univariate and multivariate cox regression analysis, we found 6 informative variables (age, primary site, tumor size, grade, AJCC stage, and surgery) as independent prognostic factors for osteosarcoma in adults. Age is a well-known prognostic factor for many tumors factor.^[22]^ This study came to the same conclusion that elderly patients have a poorer prognosis. In previous studies, tumor location was reported as the most important prognostic factor in patients with osteosarcoma.^[23–28]^ This study shows that tumor location affects survival in patients with osteosarcoma. Previous studies have shown that tumor size is also one of the key indicators of survival and prognosis in patients with osteosarcoma.^[22,29–31]^ Several previous studies have reported poorer prognosis and reduced survival in patients with larger tumors. This study suggests that tumor size is an important factor affecting the healing of patients. We also determined tumor grade and histology as independent prognostic factors in patients with osteosarcoma, which was consistent with previous studies.^[32–34]^ Tumor staging at diagnosis, several groups have reported significantly poorer survival outcomes in patients with osteosarcoma metastases.^[31,35–37]^ Consistent with these findings, we found that patients with osteosarcoma with distant metastases had a higher risk of death. Surgery has a rapid impact on survival outcomes in patients with osteosarcoma, which is consistent with previous findings.^[38–42]^ Nishida et al^[4]^ showed that the 5-year overall survival rate was 53.2% in elderly patients treated with surgery compared to 8.7% in the nonsurgical group.

Although the predictive nomograms in this study showed good predictive power, there are still some limitations that need to be considered. First, the current study only includes clinical data from patients diagnosed with osteosarcoma from 2004 to 2015 in the SEER database, not from all osteosarcoma patients. Second, because our study was retrospective, it was inevitable that some patient data were missing. Third, the current study used internal validation and lacked external validation. Therefore, it is necessary to collect further external data to validate the accuracy and reliability of the prediction model. As a next step, we will conduct further prospective studies at our institution to further validate the accuracy of this prediction model from external sources for clinical application.

In summary, age, primary site, tumor size, grade, AJCC stage and surgery were identified as independent prognostic variables for adult osteosarcoma patients. Our nomogram model provides an applicable tool with good discrimination and calibration ability, and can predict the prognosis of adult osteosarcoma patients.

## Author contributions

**Writing – review & editing:** Gaunghua Deng, Pingbo Chen.
